# The Acute Effects and Mechanism of Ketamine on Nicotine-Induced Neurogenic Relaxation of the Corpus Cavernosum in Mice

**DOI:** 10.3390/ijms24086976

**Published:** 2023-04-10

**Authors:** Ming-Wei Li, Tze-Chen Chao, Li-Yi Lim, Hsi-Hsien Chang, Stephen Shei-Dei Yang

**Affiliations:** 1 Division of Urology, Department of Surgery, Taipei Tzu Chi Hospital, Buddhist Tzu Chi Medical Foundation, New Taipei 23142, Taiwan; 2 School of Medicine, Tzu Chi University, Hualien 97004, Taiwan

**Keywords:** ketamine, *N*-methyl-D-aspartate, major pelvic ganglion, corpus cavernosum, sympathetic nerve

## Abstract

The present study aimed to investigate the acute effects and the mechanism of ketamine on nicotine-induced relaxation of the corpus cavernosum (CC) in mice. This study measured the intra-cavernosal pressure (ICP) of male C57BL/6 mice and the CC muscle activities using an organ bath wire myograph. Various drugs were used to investigate the mechanism of ketamine on nicotine-induced relaxation. Direct ketamine injection into the major pelvic ganglion (MPG) inhibited MPG-induced increases in ICP. D-serine/L-glutamate-induced relaxation of the CC was inhibited by MK-801 (*N*-methyl-D-aspartate (NMDA) receptor inhibitor), and nicotine-induced relaxation was enhanced by D-serine/L-glutamate. NMDA had no effect on CC relaxation. Nicotine-induced relaxation of the CC was suppressed by mecamylamine (a non-selective nicotinic acetylcholine receptor antagonist), lidocaine, guanethidine (an adrenergic neuronal blocker), N^w^-nitro-L-arginine (a non-selective nitric oxide synthase inhibitor), MK-801, and ketamine. This relaxation was almost completely inhibited in CC strips pretreated with 6-hydroxydopamine (a neurotoxic synthetic organic compound). Ketamine inhibited cavernosal nerve neurotransmission via direct action on the ganglion and impaired nicotine-induced CC relaxation. The relaxation of the CC was dependent on the interaction of the sympathetic and parasympathetic nerves, which may be mediated by the NMDA receptor.

## 1. Introduction

The corpus cavernosum of all species, including humans, is densely innervated by autonomic nerves. The sympathetic nervous system is important for penile erection and may contribute to the maintenance of a non-erect state [1,2]. In rat experiments, stimulating the sympathetic nervous system after removing the spinal cord below L5 resulted in corpus cavernosum relaxation [1,3,4]. These studies suggest that erection is not only a primary parasympathetic activity, but also the result of a sequence of sympathetic processes [1,3,4].

Nitric oxide (NO) is an important neurotransmitter for penile erection [5,6,7,8]. The rat corpus cavernosum expresses the α7-nicotinic acetylcholine receptor (nAChR), which modulates the neurogenic relaxation response to nicotine [9]. N^w^-nitro-L-arginine, a NO synthase (NOS) inhibitor, inhibited nicotine-induced neurogenic relaxation, which was reversed by L-arginine [6,10]. These results indicate that nicotine induced a NO-dependent neurogenic relaxation of the corpus cavernosum.

Glutamate is a major excitatory neurotransmitter in the central nervous system. The *N*-methyl-D-aspartate (NMDA) receptor (NMDAR), an ionotropic glutamate receptor family member, mediates a slow Ca^2+^ permeable component of excitatory synaptic transmission [11,12,13]. NMDARs are heterotetramers consisting of seven subunits (GluN1, GluN2 A-D, or GluN3 A-B) [14,15,16]. NMDAR can be stimulated by glycine or D-serine without activation of the glutamatergic coagonist site [14,17,18]. In brain tissue, *N*-methyl-D-aspartate (NMDA)-induced increases in cerebral blood flow were mediated by neuronally derived NO [19,20,21], and NMDAR coactivation by glutamate and D-serine increases vascular diameter in pressurized middle cerebral arteries in both endothelial and endothelium nitric oxide synthase (eNOS)-dependent mechanisms [22,23]. NMDARs have effects on multiple organs outside of the central nervous system [24]. Penile neuronal NOS, GluN1, and GluN2 NMDAR subunits are expressed in the cavernosal nerves of the corpus cavernosum and are frequently colocalized [25,26]. However, the role of NMDAR in corpus cavernosum is not well defined.

Ketamine is an antagonist of the NMDAR complex and is commonly abused by men [27,28,29]. Erectile dysfunction (ED) has been observed in male ketamine abusers [30,31] and in ketamine-treated animals [32,33]. Chen MF et al. found that ketamine acutely blocked the nAChRs on cerebral perivascular sympathetic nerves, which then reduced nicotine-induced neurogenic vasodilation [34,35]. However, the acute effects of ketamine on penile erections are unknown.

Herein, we investigated the acute effects and mechanism of ketamine on nicotine-induced neurogenic relaxation and the sympathetic–parasympathetic nerve interactions (axo–axonal interaction) of the corpus cavernosum in mice.

## 2. Results

### 2.1. Acute Effects of Ketamine Injection on Major Pelvic Ganglion

**Figure 1:**
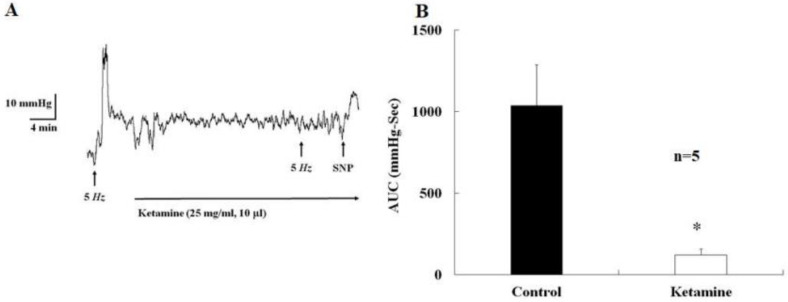
**Acute effects of ketamine on the electrical stimulation after major pelvic ganglia injection.** The representative tracing depicts activation of the major pelvic ganglia (MPG) by electrical stimulation (5 Hz) in male control mice. This increase in intra-cavernosal pressure (ICP) was abolished by ketamine injection into MPG (**A**) and partly reversed by sodium nitroprusside (SNP, 0.1 mM/5 μL) application. The decrease in ICP by ketamine (*n* = 5) injection reaches a statistical difference (**B**). Values are means ± SD. The asterisk indicates a significant difference in the paired *t*-test (* *p* < 0.05) vs. the control group.

Ketamine (25 mg/mL, 10 μL) was injected into the major pelvic ganglion of control mice using a 30-gauge needle under microscopy guidance. The area under the erectile curve of intra-cavernosal pressure was significantly reduced after the injection (Figure 1A,B, *n* = 5, *p* < 0.05).

### 2.2. Corpus Cavernosum Relaxation Induced by D-Serine and L-Glutamate

**Figure 2:**
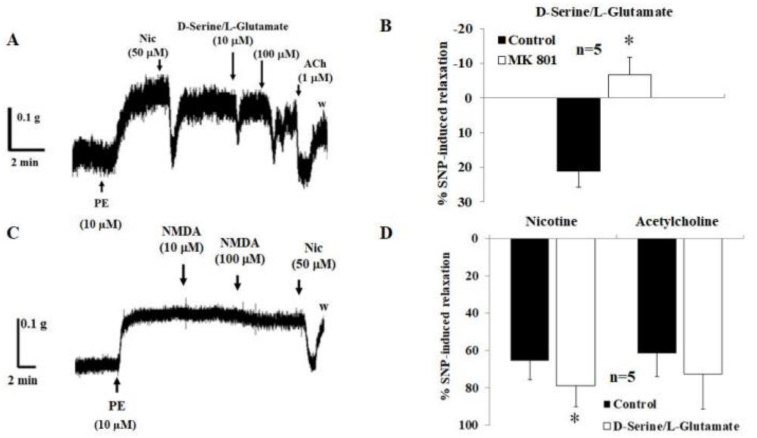
**Corpus cavernosum relaxation induced by D-serine/L-glutamate.** The representative tracing in (**A**) shows that corpus cavernosum (CC) strips are precontracted by phenylephrine (PE, 10 µM). Relaxation response induced by nicotine (Nic), D-serine/L-glutamate (10 and 100 µM), and acetylcholine (ACh, 1 µM). The D-serine/L-glutamate (100 µM)-induced relaxation was inhibited by MK-801 (10 µM, *n* = 5, *p* < 0.05 (**B**)). The representative tracing in (**C**) shows that CC relaxation was not induced by *N*-methyl-D-aspartate (NMDA, 10 and 100 µM). Nicotine-induced relaxation was significantly enhanced by pretreatment with D-serine/L-glutamate (100 µM, *n* = 5), but acetylcholine-induced relaxation was not enhanced by pretreatment with D-serine/L-glutamate (**D**). The drug-induced CC relaxation was estimated as the percentage of sodium nitroprusside (SNP, 0.1 mM)-induced maximum relaxation. *n*, number of experiments. Values are means ± SD. Asterisks indicate a significant difference in the paired *t*-test (* *p* < 0.05) vs. the control group.

Nicotine (50 µM), D-serine/L-glutamate (10 and 100 µM), and acetylcholine (1 µM) all reduced the active muscle tone of corpus cavernosum caused by phenylephrine (10 µM) (Figure 2A). This relaxation was significantly abolished by MK-801 (10 µM, Figure 2B, *n* = 5, *p* < 0.05). *N*-methyl-D-aspartate (10 and 100 µM, Figure 2C) did not induce relaxation of corpus cavernosum strips. Pre-treatment with D-serine/L-glutamate (100 µM) significantly enhanced the nicotine-induced relaxation of the corpus cavernosum (Figure 2D, *n* = 5, *p* < 0.05), but had no effect on acetylcholine-induced relaxation (Figure 2D, *n* = 5, *p* > 0.05).

### 2.3. Effects of the N-methyl-D-aspartate Receptor Inhibitor MK-801 and Ketamine on Nicotine-Induced Relaxation

**Figure 3:**
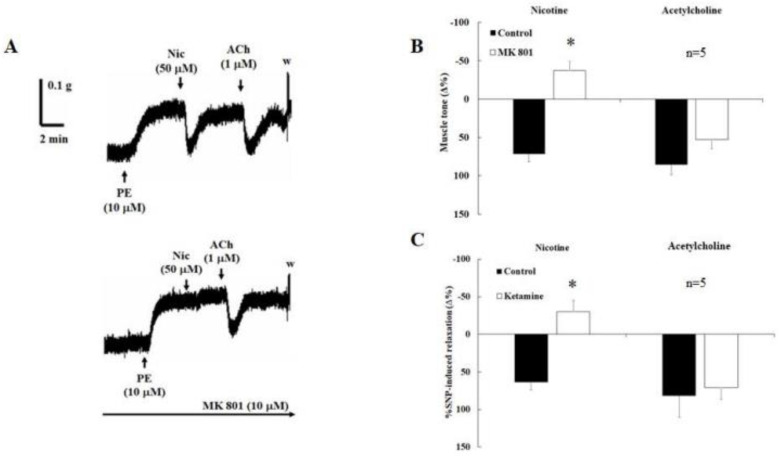
**Effect of MK-801 and ketamine on corpus cavernosum relaxation**. The representative tracing in (**A**) shows that the corpus cavernosum (CC) was precontracted by phenylephrine (PE, 10 µM). (**B**) Nicotine (Nic, 50 µM) induced CC relaxation, which was inhibited by MK-801 (10 µM) but not acetylcholine (ACh, 10 µM). (**C**) Summary data showing the effects of MK-801 on CC strips (*n* = 5, *p* < 0.05). The relaxation response induced by nicotine (50 µM) was inhibited by ketamine (10 µM, *n* = 5, *p* < 0.05). The ACh- and nicotine-induced CC smooth muscle relaxation were estimated as a percentage of sodium nitroprusside (SNP, 0.1 mM)-induced maximum relaxation. *n*, number of experiments. Values are means ± SD. Asterisks indicate a significant difference in the paired *t*-test (* *p* < 0.05) vs. the control group.

Nicotine (50 µM) and acetylcholine (1 µM) both reduced the active muscle tone of the corpus cavernosum caused by phenylephrine (10 µM) (Figure 3A). Nicotine-induced relaxation was significantly abolished by MK-801 (10 µM, Figure 3A,B, *n* = 5, *p* < 0.05) and ketamine (10 µM, Figure 3C, *n* = 5, *p* < 0.05). Acetylcholine-induced relaxation was not inhibited by MK-801 (10 µM, Figure 3A,B, *n* = 5, *p* > 0.05) and ketamine (10 µM, Figure 3C, *n* = 5, *p* > 0.05).

### 2.4. Effects of Various Drugs on Nicotine-Induced Relaxation

**Figure 4:**
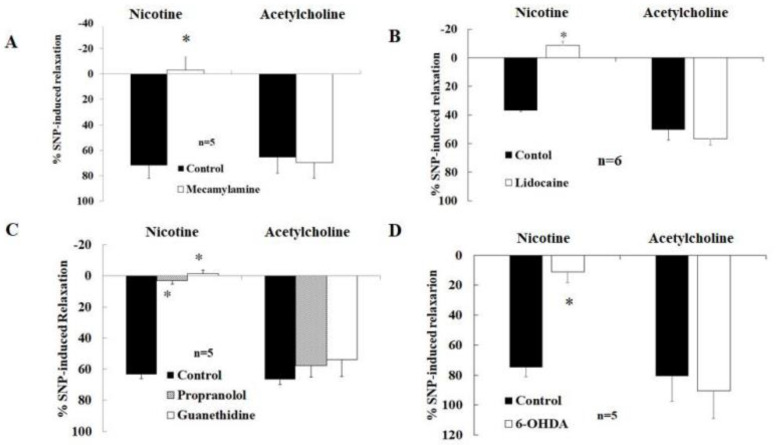
**Role of the sympathetic nerve on nicotine-induced corpus cavernosum (CC) neurogenic relaxation.** The active muscle tone of the CC was induced by phenylephrine (PE, 10 µM). Nicotine (50 µM)-induced relaxation was inhibited by pretreatment with mecamylamine (10 µM, *n* = 5, *p* < 0.05, (**A**)), lidocaine (10 µM, *n* = 6, *p* < 0.05, (**B**)), propranolol (10 µM, *n* = 5, *p* < 0.05, (**C**)), and guanethidine (10 µM, *n* = 5, *p* < 0.05, C). In addition, nicotine-induced relaxation was inhibited by 6-hydroxydopamine (6-OHDA), causing chemical denervation (*n* = 5, *p* < 0.05, (**D**)). Acetylcholine (ACh)-induced relaxation was not affected by pretreatment drugs. ACh- and nicotine-induced CC smooth muscle relaxation were estimated as a percentage of sodium nitroprusside (SNP, 0.1 mM)-induced maximum relaxation. *n*, number of experiments. Values are means ± SD. Asterisks indicates a significant difference in the paired *t*-test (* *p* < 0.05) vs. the control group in (**A**,**B**,**D**). Asterisk indicate a significant difference in Bonferroni post-tests following ANOVA (* p < 0.05) vs. control group in (**C**).

**Figure 5:**
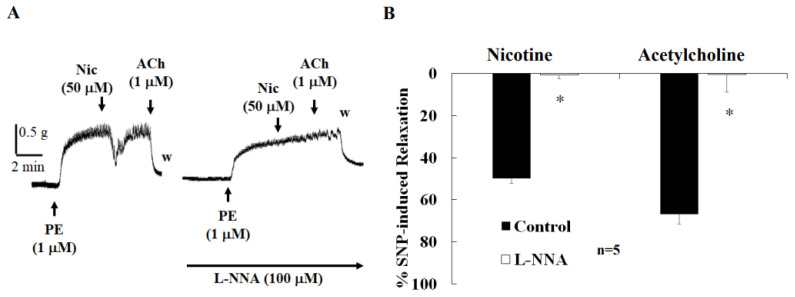
**Effect of nitric oxide synthase inhibitor N^G^-nitro-L-arginine on corpus cavernosum relaxation**. The representative tracing in (**A**) shows that the corpus cavernosum (CC) is precontracted by phenylephrine (PE, 10 µM). The relaxation induced by nicotine (50 µM) and acetylcholine (ACh, 10 µM) was inhibited by N^G^-nitro-L-arginine (L-NNA, 100 µM). (**B**) Summary data showing the effects of L-NNA on corpus cavernosum (CC) strips (*n* = 5, *p* < 0.05). ACh- and nicotine-induced CC smooth muscle relaxation were estimated as a percentage of sodium nitroprusside (SNP, 0.1 mM)-induced maximum relaxation. *n*, number of experiments. Values are means ± SD. The asterisk indicates a significant difference in the paired *t*-test (* *p* < 0.05) vs. the control group.

Nicotine (50 µM) reduced the active muscle tone of the corpus cavernosum caused by phenylephrine (10 µM). This relaxation was significantly abolished by mecamylamine (10 µM, Figure 4A, *n* = 5, *p* < 0.05). However, mecamylamine (10 µM) did not inhibit acetylcholine (1 µM)-induced relaxation of corpus cavernosum strips (Figure 4A, *n* = 5, *p* > 0.05). Lidocaine (10 µM) significantly inhibited nicotine-induced corpus cavernosum relaxation (*n* = 6, *p* < 0.05), but had no effect on acetylcholine (1 µM)-induced corpus cavernosum relaxation (Figure 4B, *n* = 6, *p* > 0.05). In addition, propranolol (10 µM) significantly inhibited nicotine-induced corpus cavernosum relaxation (Figure 4C, *n* = 5, *p* < 0.05). Similar results were observed when guanethidine (10 µM), a drug that inhibits the release of norepinephrine, was administered (Figure 4C, *n* = 5, *p* < 0.05). However, neither propranolol (10 µM) nor guanethidine (10 µM) had an effect on acetylcholine (1 µM)-induced relaxation of corpus cavernosum strips (Figure 4C, *n* = 5, *p* > 0.05). Nicotine (50 µM)-induced relaxation of active muscle tone caused by phenylephrine (10 µM) was almost completely abolished by chemical denervation with 6-hydroxydopamine (*n* = 5, *p* < 0.01, Figure 4D), whereas acetylcholine (1 µM)-induced relaxation was unaffected (Figure 4D, *n* = 5, *p* > 0.05). N^w^-nitro-L-arginine (100 µM) significantly reduced both nicotine-induced (Figure 5A,B, 49.8 ± 2.3 vs. 0.6 ± 1.9, *n* = 5, *p* < 0.05) and acetylcholine (1 µM)-induced corpus cavernosum relaxation (Figure 5A,B, *n* = 6, *p* < 0.05).

## 3. Discussion

**Figure 6:**
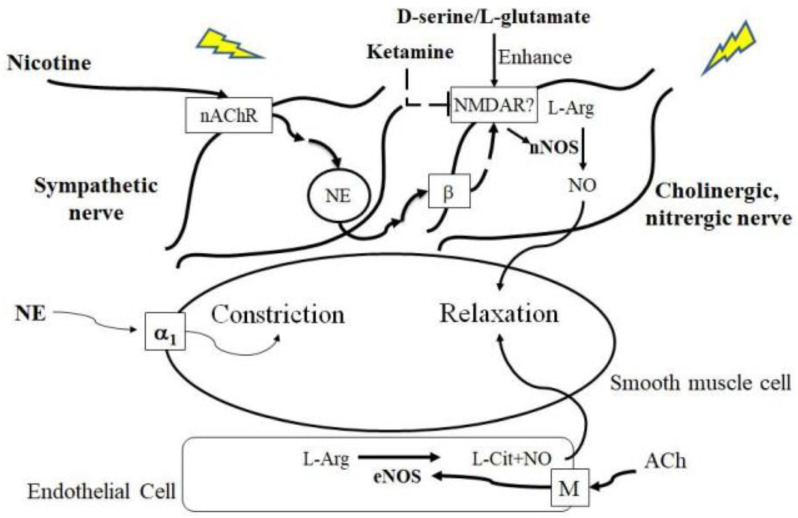
Mechanisms of ketamine on nicotine-induced neurogenic relaxation and sympathetic–parasympathetic nerve interaction in the corpus cavernosum.

The impact of ketamine on nicotine-induced neurogenic relaxation and sympathetic–parasympathetic nerve interaction (axo–axonal interaction) in the corpus cavernosum is illustrated in Figure 6. Nicotine acts on adrenergic nerve terminals, causing norepinephrine to be released, which then acts on beta-adrenergic receptors in cholinergic nerve terminals. Excitation of beta-adrenergic receptors facilitates NMDAR action, which enhances the transformation of L-arginine to NO via nNOS. Finally, NO causes the corpus cavernosum to relax. Furthermore, the interaction of nAChR and NMDA in this mechanism is dependent on intact sympathetic nerves.

Ketamine had a direct effect on the ganglions. Ketamine injection into the major pelvic ganglion decreased the area under the erectile curve of intra-cavernosal pressure significantly, whereas sodium nitroprusside (NO donor) injection into the corpus cavernosum reversed this effect (Figure 1). Voltage-dependent ion channels are critical in the generation and propagation of nerve impulses [36,37]. Therefore, the blockade of Na^+^ channels by ketamine, which reduces their excitability, may play an important role in the complex mechanism of its action during anesthesia [38,39,40]. The major pelvic ganglion receives input from the hypogastric nerve (a sympathetic nerve) and the pelvic nerve (a parasympathetic nerve) [41,42]. In addition, glutamate R1 subunits have been found in the major pelvic ganglion [42,43]. The dorsal penile nerve and major pelvic ganglion control reflexive erections via glutamatergic transmission [42,43]. NMDAR subunits are expressed in the cavernosal nerves of the corpus cavernosum [25,26]. Thus, ketamine inhibited cavernosal nerve neurotransmission by acting directly on ganglions that may bind to NMDAR.

Ketamine is an NMDAR antagonist. It binds to the allosteric phencyclidine (PCP) site that is located within the channel pore of the NMDAR. The binding affinity of ketamine to the PCP binding site has been reported to be between 0.18 and 3.1 μM in the presence of Mg^2+^ [44]. To our knowledge, the current study is the first to inject ketamine into the major pelvic ganglion. In addition, our study inhibited nicotine-induced corpus cavernosum relaxation with 1~100 μM of ketamine in a tissue bath (data not presented in this paper). Therefore, we selected the low level of 0.45 μM (25 mg/mL/10 μL/25 g mice, mimic serum ketamine concentration) of ketamine to inhibit NMDAR. Different ketamine doses had the same/similar effects on erectile function, with dose-dependent effects.

NMDAR may be involved in penile erection. NMDAR activity was activated by D-serine/L-glutamate treatment, causing corpus cavernosum relaxation (Figure 2A). This relaxation was inhibited by MK-801, proving that the NMDAR was activated by D-serine/L-glutamate (Figure 2B). NMDA, on the other hand, was unable to induce corpus cavernosum relaxation on its own (Figure 2C). Previous research found NMDAR subunits in the rat prostate and penis [26,45], and D-serine modulates the neurogenic relaxation of the rat corpus cavernosum [26,46]. D-serine is a synaptic receptor coagonist [47]. In our study, D-serine by itself was unable to induce corpus cavernosum relaxation. However, NMDAR activation requires glutamate and a coagonist, the nature of which and its impact on NMDAR physiology remain unknown [47]. Pretreatment with MK-801 and ketamine inhibited the nicotine-induced relaxation of the corpus cavernosum, indicating that NMDAR is involved in the nicotine-induced relaxation mechanism (Figure 3). This hypothesis is supported further by the inhibitory effects of MK-801 and ketamine on corpus cavernosum relaxation (Figure 3).

An intact sympathetic nervous system is important for penile erection. Nicotine-induced relaxation of the corpus cavernosum was suppressed by pretreatment with mecamylamine (a non-selective, non-competitive antagonist of the nAChRs), lidocaine (a nerve blocker), and guanethidine (an adrenergic neuronal blocker) (Figure 4). These findings are consistent with previous research showing that activation of the sympathetic nerves of the superior cervical ganglion origin induced by electrical depolarization and topical nicotine increased the basilar arterial blood flow [48,49] via activation of nicotinic agonists of nAChR located on the perivascular sympathetic nerves. Therefore, nicotine-induced neurogenic relaxation in the corpus cavernosum is similar to that in the cerebral artery. Norepinephrine is released upon activation of nAChR located on sympathetic nerves by nicotinic agonists, causing relaxation of the corpus cavernosum.

Conversely, acetylcholine-induce relaxation was not inhibited by mecamylamine, lidocaine, or guanethidine (Figure 4). These relaxations are endothelial-dependent, mediated partly by NO and other substances [2,50]. This is supported by the finding that nicotine-induced relaxation of the corpus cavernosum was almost completely blocked when pretreated with 6-hydroxydopamine and guanethidine (Figure 4). Since guanethidine and 6-hydroxydopamine significantly affect nicotine-induced neurogenic relaxation of the corpus cavernosum, their blockade of nicotine-induced vasorelaxation was not due to a possible local anesthetic effect or an alteration in smooth muscle reactivity. Hence, the axo–axonal interaction mechanism in the corpus cavernosum was dependent on an intact sympathetic nerve.

NO is the key neurotransmitter in the principal pathway of penile erection [5,6]. Basal NO released from endothelial cells inhibits contractions in the mouse corpus cavernosum [27]. In rats, unilateral carvenosal denervation resulted in decreases in intra-cavernosal pressure and NOS fibers [6]. Furthermore, by inhibiting nNOS, muscarinic receptors may modulate NO synthesis in nitrergic nerves [51,52]. In the current study, nicotine- and acetylcholine-induced corpus cavernosum relaxation was inhibited by N^w^-nitro-L-arginine (Figure 5). These findings suggest nicotine-induced neurogenic relaxation in the corpus cavernosum is NO dependent. It was reported that nicotine acted on the nAChRs located on the nitrergic nerves, thereby evoking the release of NO from these nerve terminals and inducing corpus cavernosum relaxation in rabbits [10]. Moreover, α7-nAChR was found in rat corpus cavernosum and was shown to modulate the neurogenic relaxation response to nicotine [9]. This is supported by the discovery that acetylcholine induces endothelial and NO-dependent relaxation in the corpus cavernosum. Furthermore, the neurogenic relaxations of the corpus cavernosum are mediated by NO synthesized from L-arginine in nerve terminals, and this nerve originates from ganglia near the corpus cavernosum rather than directly from the pelvic nerve plexus [53].

In real-world settings, erectile dysfunction is frequently observed among ketamine abusers [30]. Ketamine-related erectile dysfunction may be partly explained by the current findings that ketamine inhibits cavernosal nerve neurotransmission and impairs corpus cavernosum relaxation. In the future, new treatments targeting the NMDA receptor or its associated pathways could be developed to treat erectile dysfunction in these patients. Despite the importance of an intact sympathetic nervous system for penile erection, as demonstrated in this in vitro study, sympathomimetics do not play a role in the current treatment of erectile dysfunction. On the contrary, sympathomimetics cause vasoconstriction and are used to treat priapism [54]. Further investigation into the transmission pathways is necessary to develop new treatment strategies for erectile dysfunction.

NMDAR plays an important role in this axo–axonal transmitting pathway. It is known to be present in cavernosal nerves [25,26]. The limitation of this study is that it does not investigate the presence of NMDAR on the cavernosal nerve. Perhaps the mechanism of NOS stimulation is much more complex. The current study is also limited by its small sample size. We chose a smaller sample size in accordance with the 3Rs of reduction principles established by the Committee on the Care and Use of Laboratory Animals. To strengthen the credibility of our findings, we repeated the evaluation of nicotine- and acetylcholine-induced corpus cavernosum relaxation with drugs that had similar effects, such as MK-801 and ketamine for the effects on NMDAR, and guanethidine and 6-hydroxydopamine for the effects on sympathetic nerves. Furthermore, this study used C57BL/6 mice, which have the same genetic background.

## 4. Material and Methods

### 4.1. Intra-Cavernosal Pressure (ICP) Measurement

This experiment was approved by our institute’s Laboratory Animal Care and Use Committee. Five-week-old male C57BL/6 (B6) mice were kept under controlled light (12 h light/dark cycles from 7:00 a.m. to 7:00 p.m.) and temperature (21 °C to 23 °C) conditions. Mice were anesthetized with urethane (500 mg/kg) and chloralose (50 mg/kg). Following anesthesia, an incision was made from the white line of the lower abdomen to the bladder. To expose the major pelvic ganglia, connective tissues were removed with extreme caution to avoid bleeding. The cavernosal nerve and major pelvic ganglia were hooked up to an electrode wire that was connected to an electrical stimulator (Grass, SD9J). A 30-gauge needle was attached to a polyethylene (PE) 10 catheter filled with normal saline containing heparin (100 IU). The needle was carefully inserted into the corpus cavernosum of the penis, while the end of the PE 10 catheter was connected to a pressure sensor port. The ICP was measured using an BIOPAC MP36 (Biopac Systems Inc., Santa Barbara, CA, USA) and Biopac Student Lab (BSL) 3.7.3 software (Biopac Systems Inc., Santa Barbara, CA, USA). The ICP was measured for 60 s while the cavernous nerve was electrically stimulated (ES, 2–10 Hz, 50 msec, and 2.5 V). The total ICP was calculated as the area under the erectile curve (AUC) from the start of cavernous nerve stimulation until the ICP returned to baseline.

### 4.2. Tissue Preparation

After anesthesia with urethane (500 mg/kg, intraperitoneal (ip)) and chloralose (50 mg/kg, ip), the B6 mice were sacrificed by cervical dislocation. The corpus cavernosum was dissected and placed in oxygenated (95% O_2_ and 5% CO_2_) Krebs’ bicarbonate solution at 4 °C. The Krebs’ bicarbonate solution (in mM) contained NaCl (117), NaHCO_3_ (25), KCl (4.7), CaCl_2_ (2.5), MgSO_4_ (1.2), KH_2_PO_4_ (1.2), glucose (11.1), and calcium disodium ethylenediamine tetraacetate (EDTA) (0.023).

### 4.3. Tissue Bath Wire Myography

The corpus cavernosum was dissected and cleaned of surrounding tissue under a dissecting microscope, then mounted on a stainless-steel rod and a platinum wire in a tissue bath containing 20 mL Krebs’ bicarbonate solution, equilibrated with 95% O_2_ and 5% CO_2_ and maintained at 37 °C. An isometric transducer (FT03C; Grass) was used to measure tension changes.

The corpus cavernosum strips were equilibrated in the Krebs’ bicarbonate solution for 60 min before being mechanically stretched to a resting tension of 0.2 gm. Step 1: After equilibration, the resting muscle tone of corpus cavernosum strips was altered by cumulative applications of phenylephrine (0.001~10 µM). Step 2: The corpus cavernosum strips were pre-contracted with phenylephrine (10 µM), and then relaxation effects were induced by acetylcholine (1 µM) and nicotine (50 µM) using a BIOPAC MP36 (Biopac Systems Inc., Santa Barbara, CA, USA) and Biopac Student Lab (BSL) 3.7.3 software (Biopac Systems Inc., Santa Barbara, CA, USA), respectively. There were 45 min of washing with Krebs’ bicarbonate solution between steps 1 and 2. Step 3: After washing, lidocaine (1 µM), propranolol (10 µM), guanethidine (10 µM), and N^G^-nitro-L-arginine (100 µM) were applied, followed by phenylephrine (10 µM) 15 min later to induce active muscle contraction, and the acetylcholine- and nicotine-induced relaxation were recorded. Step 4: After washing, corpus cavernosum strips were pre-contracted with phenylephrine (10 µM), and then relaxation effects were induced by sodium nitroprusside (SNP, 0.1 mM). This myography study utilized only one isolated corpus cavernosum strip per animal. The acetylcholine- and nicotine-induced corpus cavernosum smooth muscle relaxations were expressed as a percentage of the sodium nitroprusside (SNP, 0.1 mM)-induced maximum relaxation.

### 4.4. Chemical Denervation with 6-hydroxydopamine

As previously reported [55], chemical denervation of the sympathetic nerves of the corpus cavernosum was performed in vitro by incubating the artery with 6-hydroxydopamine. Since 6-hydroxydopamine is extremely susceptible to oxidation at neutral and alkaline pHs, glutathione (40 μM) was added to the unbuffered Krebs’ bicarbonate solution (NaHCO_3_ was omitted from the solution) to slow its oxidation. The corpus cavernosum strips were incubated twice for 30 min each in Krebs’ bicarbonate solution (37 °C) containing 6-hydroxydopamine (4 mM), with a 20 min interval in normal Krebs’ bicarbonate solution. After chemical denervation, followed by phenylephrine (10 µM) to induce active muscle contraction, acetylcholine- and nicotine-induced relaxation were recorded.

### 4.5. Drugs

The following chemicals were used: NaCl (117 mM), NaHCO_3_ (25 mM), KCl (4.7 mM), CaCl_2_ (2.5 mM), MgSO_4_ (1.2 mM), KH_2_PO_4_ (1.2 mM), glucose (11.1 mM), and calcium disodium ethylenediamine tetraacetate (EDTA, 0.023 mM) to form a Krebs’ bicarbonate solution for use in tissue bath wire myography. Acetylcholine (1 μM), lidocaine (10 μM), N^w^-nitro-L-arginine (100 μM), sodium nitroprusside (0.1 mM), nicotine (50 μM), phenylephrine (10 μM), guanethidine (10 μM), MK-801 (10 μM), D-serine (10 and 100 μM), L-glutamate (10 and 100 μM), and propranolol (10 μM) were used to determine the mechanism of nicotine-induced cavernous relaxation. 6-hydroxydopamine (4 mM) and glutathione (40 μM) were used in the denervation of sympathetic nerves of the corpus cavernosum. These chemicals were all purchased from Sigma-Aldrich, St. Louis, MO, USA.

### 4.6. Statistical Analysis

All data are expressed as means ± standard deviation and represented in a bar chart. To compare the differences between different strips, an ANOVA was used, followed by post hoc tests (Bonferroni) in the case of a normal distribution. A paired t-test was used to compare differences within the same strip. A *p*-value of < 0.05 was considered statistically significant (SigmaSate version 3.5 for Windows, Systat Software, San Jose, CA, USA).

## 5. Conclusions

Ketamine may induce erectile dysfunction at the ganglion level and NMDAR at the axonal terminal. The axon–axonal interaction mechanism may play a critical role in mediating corpus cavernosal relaxation during erection of the penis. This axon–axonal mechanism may interact via NMDAR.

## Data Availability

All data generated or analyzed during this study can be provided on request to and upon approval of the corresponding author, Y.S.S.-D.
